# Evaluating the trade‐off between plan complexity, dosimetric accuracy, and treatment efficiency: Role of aperture shape controller settings in HyperArc for intracranial oligometastases

**DOI:** 10.1002/pro6.70034

**Published:** 2025-12-22

**Authors:** Huipeng Meng, Yanlong Zhang, Xinrui Wang, Pengfei Liu, Weihua Zhu, Shixiong Huang, Yining Yang

**Affiliations:** ^1^ Department of Radiation Oncology Tianjin First Central Hospital, School of Medicine, Nankai University Tianjin China; ^2^ School of Medical Technology Tianjin Medical College Tianjin China; ^3^ Clinical Application Training Department Varian Medical Systems Inc. Beijing China; ^4^ Department of Radiation Oncology The Affiliated Cancer Hospital of Xiangya School of Medicine Central South University/Hunan Cancer Hospital Changsha China

**Keywords:** Aperture shape controller, dosimetry, HyperArc, intracranial oligometastases, radiotherapy, treatment efficiency

## Abstract

**Purpose:**

To evaluate the impact of different aperture shape controller (ASC) stratification strategies on the dosimetric quality and treatment efficiency of HyperArc plans for intracranial oligometastases.

**Methods:**

This retrospective study analyzed 17 patients with 1–3 intracranial oligometastases. For each patient, the HyperArc plans were reoptimized using six ASC strength settings (off, very low, low, moderate, high, and very high). Evaluations encompassed planning target volume (PTV) and organ at risk (OAR) dosimetric parameters (Paddick Conformity Index [Paddick CI], Gradient Index [GI], D_2cm_, OAR D_max_/D_mean_, Gamma passing rate), and treatment efficiency parameters (monitor units [MUs], segment number [SN], Modulation Complexity Score [MCS], Average Leaf Trajectory [ALT], and aperture irregularity [AI]).

**Results:**

No statistically significant differences were observed in the PTV and OAR dosimetric parameters or gamma passing rates among the ASC strategy groups (*p* > 0.05), indicating a minimal impact of the ASC on plan dosimetric quality. In addition, SN, MCS, and ALT demonstrated no significant intergroup differences (*p* > 0.05). However, AI improved with moderate and high ASC strength. Critically, the total MUs differed significantly among the groups (*F* = 2.904, *p* < 0.05), with high ASC strength causing significantly lower MUs than ASC‐off (*p* < 0.05), suggesting enhanced treatment efficiency.

**Conclusions:**

ASC stratification strategies do not significantly compromise plan dosimetric quality in HyperArc treatment for intracranial oligometastases; however, these can markedly optimize treatment efficiency, particularly by reducing MUs. Considering both plan complexity and treatment efficiency, a moderate or high ASC strength is recommended to maintain high‐quality radiotherapy while improving workflow efficiency.

## Graphical abstract

In HyperArc™ SRS for intracranial oligometastases, Aperture Shape Controller (ASC) settings minimally affect dosimetric quality and overall plan complexity. High ASC strengths significantly reduce Monitor Units (MUs), enhancing treatment efficiency, thereby achieving an optimal balance for clinical use.

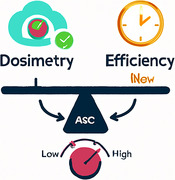

## 1 INTRODUCTION

The precise delivery of radiation, while effectively sparing organs at risk (OARs), is a central objective in modern radiotherapy.^1^, ^2^ Volumetric modulated arc therapy (VMAT) has become a standard technique that is widely adopted in clinical practice because of its capacity to generate highly conformal dose distributions.^3^, ^4^ However, the inherent modulation complexity of VMAT plans can cause increased monitor units (MUs), prolonged treatment times, and more stringent demands on the precision of multileaf collimator (MLC) movement, thereby introducing potential delivery uncertainties.^5^, ^6^ To address these challenges, several optimization tools, such as the aperture shape controller (ASC), have been integrated into treatment planning systems (TPS). The ASC penalizes excessively small or irregular MLC aperture shapes during optimization to smooth leaf trajectories and reduce aperture complexity, potentially improving plan deliverability and decreasing MUs.^7^, ^8^, ^9^ Users can select different ASC strength levels based on their clinical needs to balance plan quality and complexity.

For intracranial oligometastases, HyperArc™ technology (Varian Medical Systems; Palo Alto, CA, USA), a highly automated solution specifically designed for stereotactic radiosurgery (SRS), demonstrates significant advantages in target conformity and dose‐falloff gradients through pre‐defined non‐coplanar arc trajectories and optimized dose algorithms.^5^, ^10^, ^11^ Although HyperArc is intended to simplify SRS workflows and enhance treatment quality, its complex noncoplanar irradiation and multitarget optimization can sometimes cause high MUs and intricate MLC sequences.^5^, ^12^ Theoretically, integrating the ASC aperture‐smoothing mechanism with HyperArc technology could further reduce plan complexity and MUs while maintaining or even enhancing dosimetric advantages, thereby optimizing treatment delivery efficiency and plan robustness.^5^, ^13^

Previous research on ASC has predominantly focused on conventional VMAT plans, where the impact on plan quality (e.g., target coverage and OAR sparing) varies depending on the treatment site, ASC strength setting, and interaction with other optimization parameters, such as the convergence mode.^7^, ^14^ Certain studies have reported that excessively high ASC strengths may adversely affect dose distributions.^15^ However, only a few studies have systematically evaluated the impact of different standard ASC settings (i.e., not specifically modified strategies) on treatment plans for intracranial oligometastases within an automated HyperArc workflow, particularly concerning the trade‐off between dosimetric quality and treatment efficiency. It is crucial to elucidate the specific effects of different ASC strength levels on plan complexity, dosimetric characteristics, and treatment efficiency within the HyperArc framework for guiding more refined and individualized applications of this combined technology in clinical practice.^14^, ^16^

Therefore, we hypothesized that the application of standard ASC settings in HyperArc would reduce plan complexity and improve treatment efficiency (e.g., lower MUs) without a clinically significant trade‐off in dosimetric plan quality. We tested this hypothesis by retrospectively analyzing the comprehensive impact of different ASC stratification strategies on dosimetric parameters and treatment delivery efficiency of HyperArc plans for intracranial oligometastases. The goal was to provide direct evidence‐based guidance for clinical medical physicists on how to effectively enhance treatment efficiency while ensuring plan quality when optimizing HyperArc plans, thus enabling clinics to leverage the full potential of HyperArc automation to improve throughput and patient comfort without compromising treatment quality.

## 2 MATERIALS AND METHODS

### 2.1 Patient Data and Eligibility Criteria

This retrospective study was conducted in accordance with the ethical guidelines of the Declaration of Helsinki. The study protocol was approved by the Science and Technology Ethics Committee of Tianjin First Central Hospital (TFCH) (approval No. 20241031‐1), which waived the requirement for patient informed consent because of the retrospective nature of the analysis and the utilization of fully anonymized data. This retrospective study included imaging data from 17 patients with intracranial oligometastases (1–3 lesions) previously treated with HyperArc™ SRS. Inclusion criteria were (1) pathologically or radiologically confirmed solid tumor brain metastases with previous radiotherapy completed, (2) pre‐treatment magnetic resonance imaging (MRI) confirming one to three intracranial metastatic lesions, (3) availability of complete simulation computed tomography (CT) images and relevant clinical data sufficient for re‐planning, (4) Karnofsky Performance Status (KPS) score ≥70, and an expected survival of more than 6 months. Exclusion criteria included (1) severe artifacts in planning CT images from metallic implants (e.g., dental restorations) precluding accurate OAR delineation, and (2) maximum volume of a single metastatic lesion exceeding 25 cm^3^.

### 2.2 Equipment and Software

The patient simulations were performed using a Philips Brilliance Big Bore 16‐slice CT scanner (Philips Healthcare; Amsterdam, The Netherlands). Treatment planning and optimization were conducted on the Varian Eclipse™ TPS (version 15.6; Varian Medical Systems, Palo Alto, CA, USA). The HyperArc™ planning module (version 15.5 integrated within Eclipse v15.6) was utilized for automated non‐coplanar plan generation. All plans were designed for delivery on a Varian TrueBeam™ 2.7 linear accelerator (Varian Medical Systems, Palo Alto, CA, USA). Plan dose verification involved the accelerator's integrated aSi‐1000 amorphous silicon electronic portal imaging device (EPID), with gamma analysis performed using the Portal Dosimetry™ software (version 15.6).

### 2.3 Treatment Planning and ASC Strategies

For each patient, the HyperArc plans were re‐optimized using six pre‐defined ASC strategies, maintaining the original target delineations, OAR constraints, and a prescription dose of 27 Gy in three fractions (9 Gy per fraction). The ASC strategies consisted of ASC‐off (nASC) and five strength levels: very low (vlASC), low (loASC), moderate (mASC), high (hASC), and very high (vhASC). The resulting plans were named Plan_nASC, Plan_vlASC, Plan_loASC, Plan_mASC, Plan_hASC, and Plan_vhASC. All plans were normalized such that 98% of the planning target volume (PTV) received 27 Gy.

Plans were generated using the HyperArc module with a standardized optimization template. For a given case, all optimization parameters, except the ASC strength, were kept identical. Key design parameters included calculation and optimization of grid resolution of 1.25 mm, Acuros™ XB (AXB) dose calculation algorithm (version 15.6), 6 MV flattening filter‐free (FFF) beam, and a dose rate of 1400 MU/min. HyperArc automatically generates four standard non‐coplanar arcs with system‐optimized collimator angles. Minor adjustments to the optimization objectives within the template were permitted to ensure target coverage. Convergence mode (CM) was enabled for all plans to ensure optimal convergence, particularly at higher ASC strengths.

### 2.4 Dosimetric Evaluation Metrics

#### 2.4.1 PTV Dosimetric Parameters

The following parameters were evaluated according to the Radiation Therapy Oncology Group (RTOG) guidelines and relevant literature^17^, ^18^, ^19^:

Conformity Index (Paddick CI): *CI = (TV*_PIV_ */TV)×(TV*_PIV_ */V*_RX_), where TV_PIV_ is the target volume covered by the prescribed isodose, TV is the total target volume, and V_Rx_ is the total volume covered by the prescribed isodose. A value closer to 1 indicates better conformity.

Gradient Index (GI): GI = *V*_0.5RX_/*V*_RX_, where V_0.5Rx_ is the volume covered by 50% of the prescription isodose. A smaller GI indicates a steeper dose fall‐off.

D_2cm_: Maximum point dose to normal tissue 2 cm from the PTV edge.

#### 2.4.2 OAR Dosimetric Parameters

The maximum point dose (D_0.03cc_) to the brainstem, bilateral optic nerves, optic chiasm, bilateral lenses, bilateral eyes, and the mean whole‐brain dose (brain D_mean_) were recorded.

#### 2.4.3 Isodose Distribution Assessment

Visual inspection of isodose distributions on representative axial, sagittal, and coronal CT slices focused on the conformality of high‐dose (100% prescription isodose) and low‐dose (50% prescription isodose) and OAR sparing.

#### 2.4.4 Plan Dose Verification

The EPID‐based field dose verification assessed the agreement between the calculated and delivered doses using the gamma analysis^20^, ^21^ (3%/2 mm, 2%/2 mm, and 1%/1 mm criteria).

All dosimetric parameters were batch‐extracted using an in‐house Python‐based platform, Radiotherapy Dosimetry Omics Platform (RadDOP).

### 2.5 Plan Complexity and Treatment Efficiency Metrics

The following metrics were assessed to quantify plan complexity and treatment efficiency:

**Segment Number (SN)**: Total segments per arc.

**Modulation Complexity Score (MCS)** ^22^, ^23^:

(1) $$MCS_{arc}=\sum_{i=1}^{I-1}\left[\frac{\left(AAV_{cp_{i}}+AAV_{cp_{i+1}}\right)}{2}\times \frac{\left(LSV_{cp_{i}}+LSV_{cp_{i+1}}\right)}{2}\times \frac{MU_{CP_{i,i+1}}}{MU_{arc}}\right]$$

The MCS was calculated per arc, where for $MCS_{arc}$ as the current arc, I is the total number of control points, $AAV_{cp_{i}}$ is the MLC area variability coefficient, $LSV_{cp_{i}}$ is the leaf sequence variability coefficient, $MU_{CP_{i,i+1}}$ is the MUs for the adjacent control points, and $MU_{arc}$ is the MUs for the current arc. A higher MCS value indicates a simpler plan.

**Aperture Irregularity (AI)** ^23^

(2) $$AI_{i}=\frac{AP_{i}^{2}}{AA_{i}\cdot 4\pi }$$

AI was calculated per control point, where $AP_{i}$ is the aperture perimeter, and $AA_{i}$ is the aperture area. A higher AI value indicates more irregular and complex apertures.

**Average Leaf Trajectory (ALT)** ^24^, ^25^: ALT refers to the average travel distance of all MLCs. A smaller ALT value indicated a simpler plan.

**Monitor Units (MUs)**: Total MUs for the plan. Lower MUs are generally correlated with higher efficiency.

The complexity metrics (SN, MCS, ALT, and AI) were extracted or calculated using an in‐house C#ESAPI script. These custom tools were internally validated against manual calculations and commercial software outputs to ensure accuracy.

### 2.6 Statistical Analysis

SPSS version 26.0 (IBM Corp., Armonk, NY, USA) was used for all statistical analyses. Levene's test was used to assess the homogeneity of variance. One‐way analysis of variance (ANOVA) (or Welch's ANOVA if the variances were heterogeneous) was used to compare the groups. Post‐hoc least significant difference (LSD) tests were used for pairwise comparisons if the ANOVA results were significant. A two‐tailed *p* < 0.05 was considered statistically significant.

## 3 RESULTS

All 17 patients were successfully replanned using the six ASC strategies, with all generated plans meeting the predefined clinical dosimetric requirements.

### 3.1 PTV Dosimetric Parameters

**FIGURE 1:**
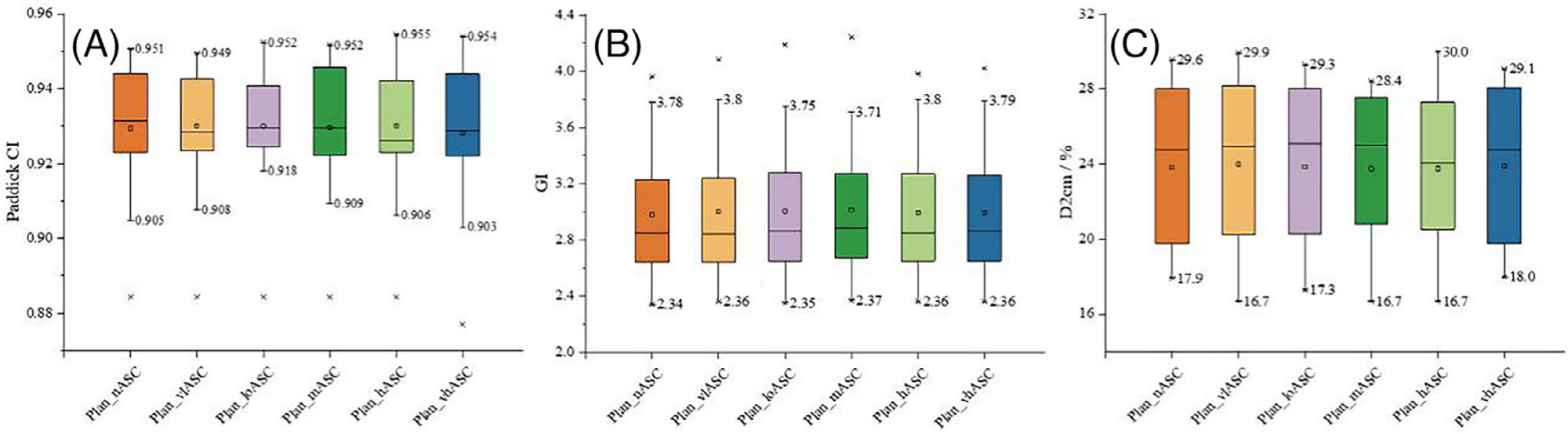
Comparison of PTV dosimetric parameters (Paddick CI, GI, D_2cm_) for HyperArc plans under different ASC strategies. All data points, including outliers, were included in the statistical analysis. *(Boxplot: central line = median; box edges = interquartile range; whiskers = min/max non‐outlier values; crosses = outliers* *.)* PTV, planning target volume; Paddick CI, Paddick conformity index; GI, gradient index; ASC, aperture shape controller.

No statistically significant differences were found between the six ASC strategy groups for Paddick CI (*F*(5, 96) = 0.03, *p* = 1.000), GI (*F*(5, 96) = 0.01, *p* = 1.000), or D_2cm_ (F(5, 96) = 0.01, *p* = 1.000) (Figure 1). Observational trends suggested a slightly more favorable GI and D_2cm_ with moderate ASC strength (Plan_mASC), whereas a very high ASC strength (Plan_vhASC) demonstrated a relatively poorer Paddick CI. Overall, the ASC settings minimally affected the dosimetric quality of the PTV.

### 3.2 OAR Dosimetric Parameters

**FIGURE 2:**
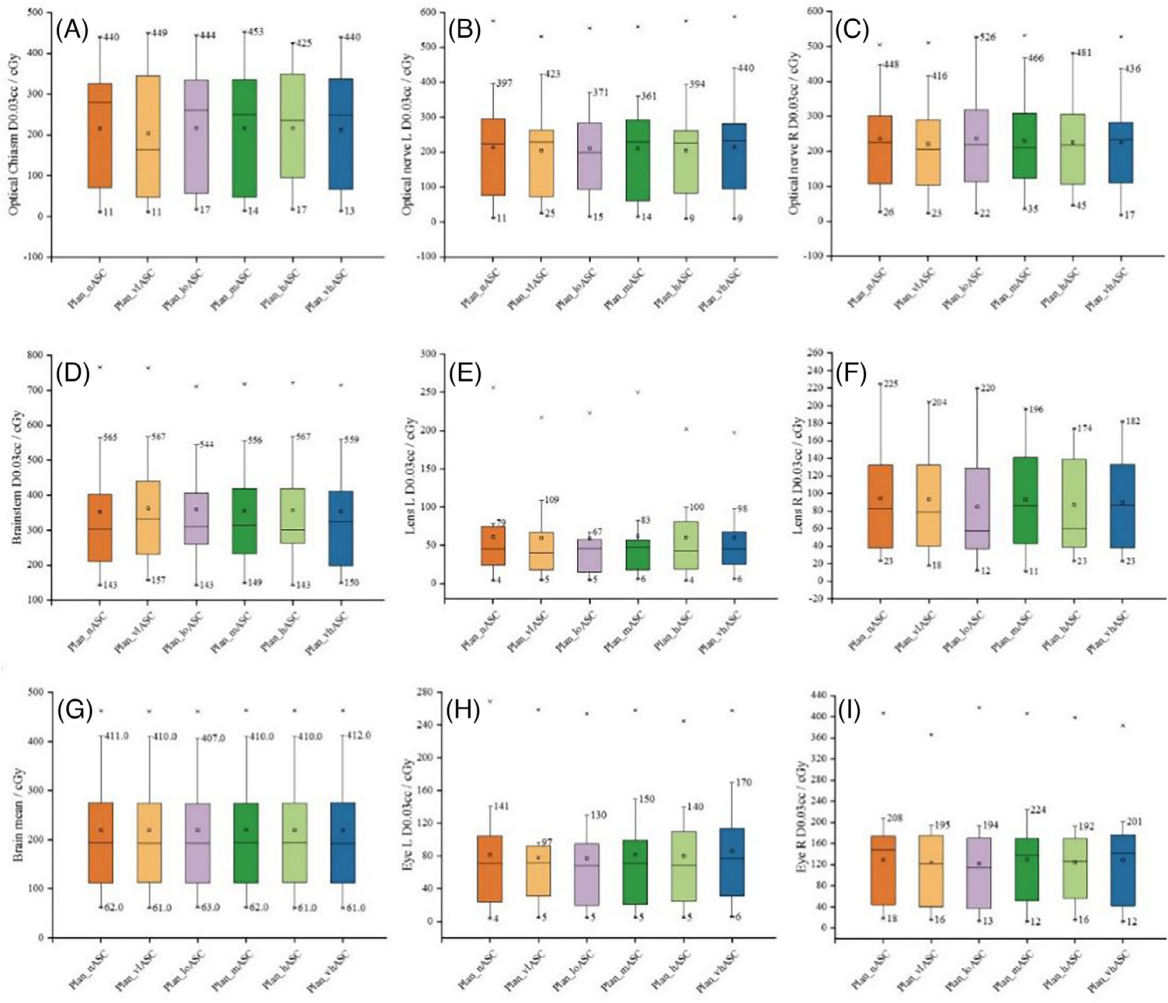
Comparison of OAR dosimetric parameters for HyperArc plans under different ASC strategies. OAR, organs at risk; ASC, aperture shape controller

Doses to all OARs (D_0.03cc_ for brainstem, optic nerves, optic chiasm, lenses, eyes, and brain D_mean_) demonstrated no statistically significant differences among the six ASC groups (all *F*(5, 96) values ranged from 0.00 to 0.07, all *p* > 0.05) (Figure 2). Optimal OAR sparing was not consistently associated with any specific ASC strength. Enabling ASC did not systematically compromise OAR protection compared with ASC off (Plan_nASC).

### 3.3 Plan Complexity and Treatment Efficiency Metrics

#### 3.3.1 SN, MCS, and ALT

**FIGURE 3:**
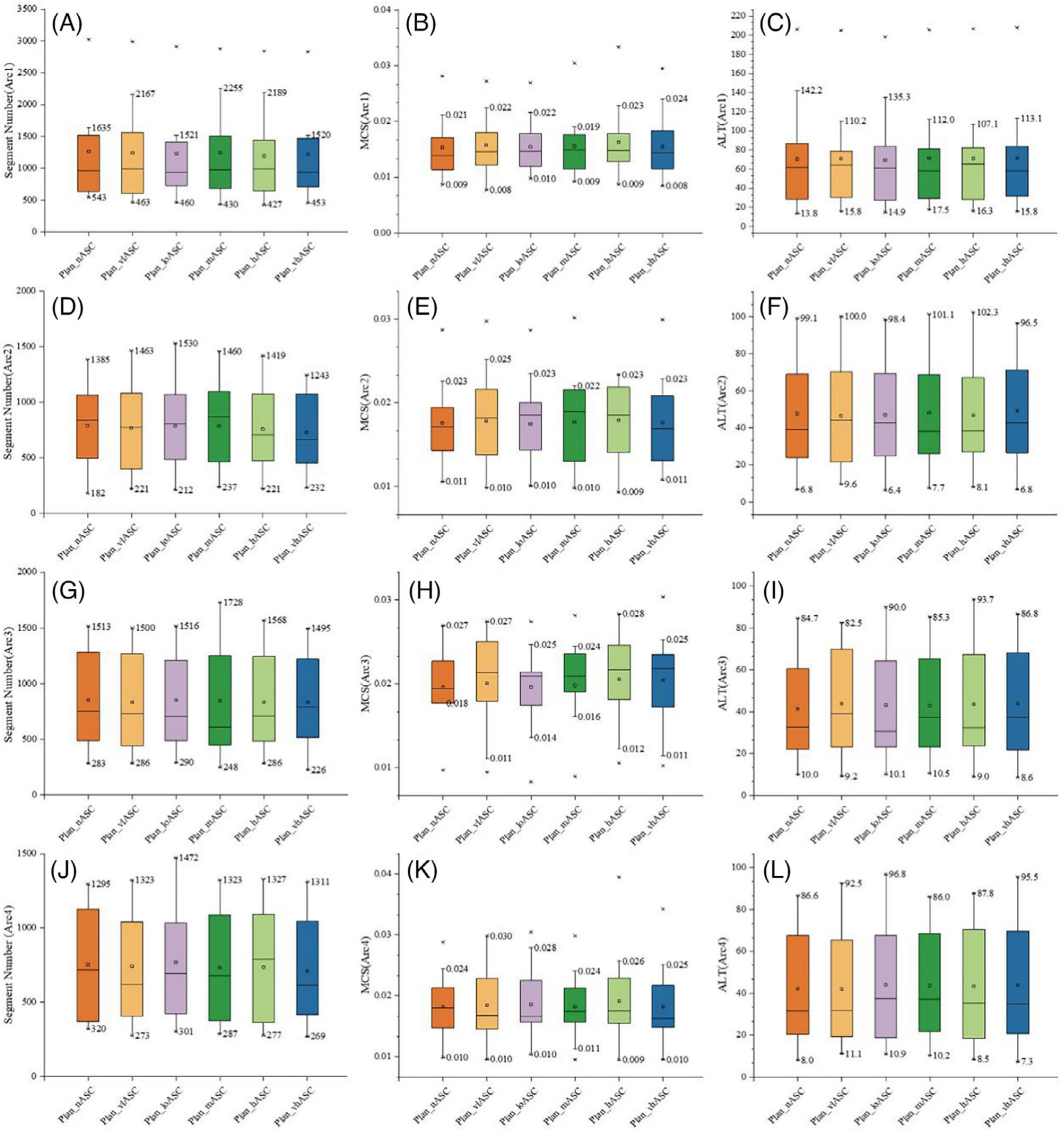
Comparison of plan complexity parameters (SN, MCS, and ALT) for HyperArc plans under different ASC strategies. SN, segment number; MCS, modulation complexity score; ALT, average leaf trajectory

No statistically significant differences were observed in SN, MCS, or ALT when comparing the six different ASC strategy groups (all *F* (5, 96) values ranged from 0.00 to 0.10, all *p* > 0.05) (Figure 3). The trends suggest that the high or very high ASC strength modes slightly reduced SN, whereas the high ASC strength mode exhibited a marginal advantage in MCS, and the very low or low ASC strength modes caused slightly lower ALT values.

#### 3.3.2 Aperture Irregularity (AI)

**FIGURE 4:**
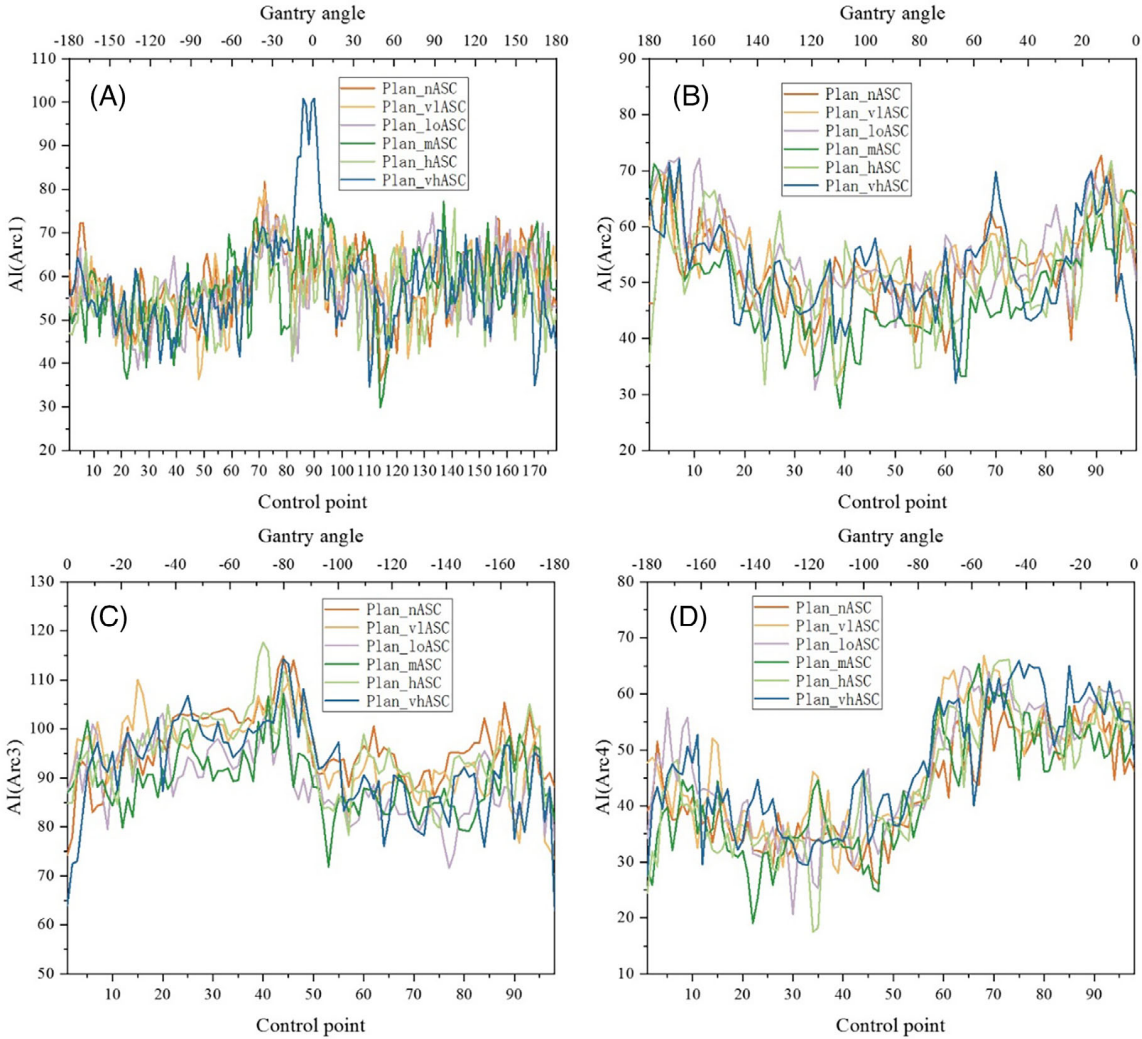
Aperture irregularity for a representative case under different ASC strategies in HyperArc plans.

In most cases, the AI values decreased with moderate (Plan_mASC) and high (Plan_hASC) ASC strengths compared to other settings (including ASC off), suggesting more regular MLC aperture shapes (Figure 4, representative case).

#### 3.3.3 Total MUs

Key findings related to total MUs were as follows. The mean (±standard deviation) MUs for different ASC strategies were Plan_nASC, 2990.7 ± 305.4; Plan_vlASC, 2940.9 ± 301.6; Plan_loASC, 2997.4 ± 270.5; Plan_mASC, 2943.5 ± 273.4; Plan_hASC, 2718.5 ± 244.9; and Plan_vhASC, 2821.6 ± 288.1.

**FIGURE 5:**
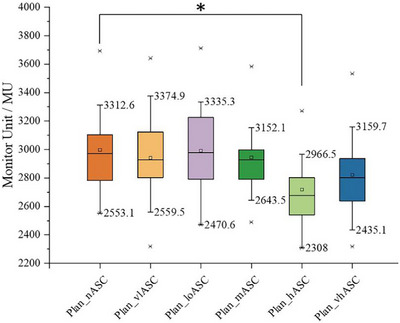
Comparison of total monitor units for HyperArc plans under different ASC strategies. ASC, aperture shape controller

A significant difference in the total MUs was observed among the ASC groups (*F* (5, 96) = 2.59, *p* = 0.030). Post‐hoc LSD tests revealed that the high ASC strength setting (Plan_hASC) yielded a clinically meaningful and statistically significant reduction in MUs compared to the ASC off setting (Plan_nASC), with an average decrease of 272.2 MUs (95% *CI*: −470.5 to −87.3 MUs, *p* = 0.005). Although the MUs for Plan_hASC were also lower than those for very high ASC strength (Plan_vhASC), this difference was not statistically significant (*p* > 0.05). Figure 5 demonstrates the advantage of high ASC strength in reducing the total number of MUs.

Asterisks (*) indicate a statistically significant difference (*p* < 0.05) between the hASC and nASC groups.

### 3.4 Plan Dose Verification (Gamma Passing Rate)

**FIGURE 6:**
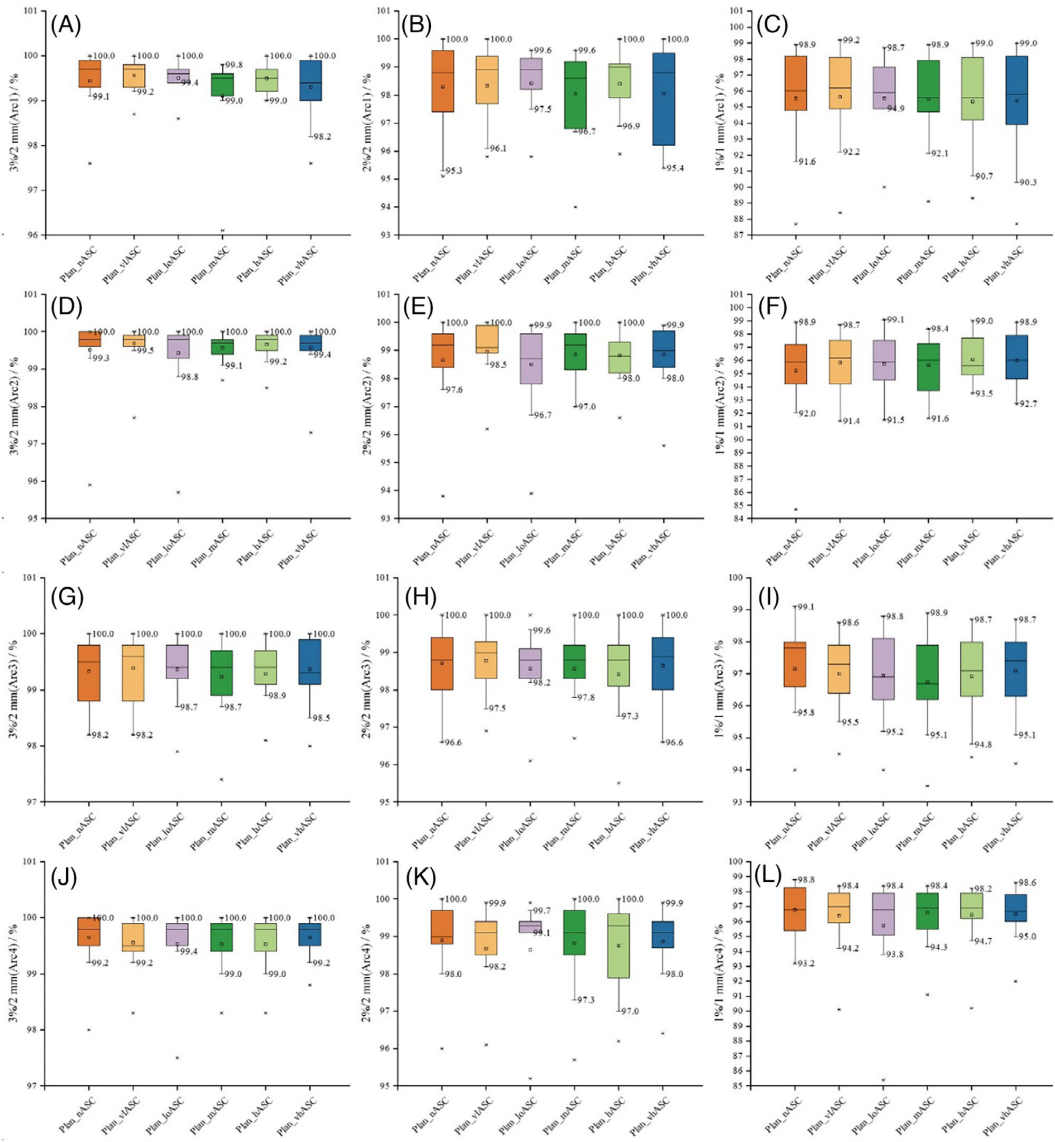
Comparison of gamma passing rates for each treatment arc under different ASC strategies. ASC, aperture shape controller

For ARC1, 3%/2 mm gamma data were not homogeneous (Levene's test, Table 1); Welch's ANOVA was used. For all other gamma analyses, no statistically significant differences in mean passing rates were found among ASC groups for 3%/2 mm, 2%/2 mm, or 1%/1 mm criteria (all *F* (5, 96) values ranged from 0.02 to 0.89, all *p* > 0.05). Trends suggested slightly higher passing rates for 3%/2 mm with high/very high ASC and for 1%/1 mm with low/moderate ASC; however, these were not significant (Figure 6). Different ASC settings did not significantly affect HyperArc plan deliverability, as measured by gamma passing rates.

**TABLE 1:** Levene's test for homogeneity of variances for gamma passing rates

Field		3%/2 mm	2%/2 mm	1%/1 mm
ARC1	*F*	2.374	1.432	0.225
	*p*	0.045^*^	0.220	0.951
ARC2	*F*	1.084	0.741	0.892
	*p*	0.119	0.595	0.490
ARC3	*F*	0.457	0.457	0.055
	*p*	0.807	0.807	0.998
ARC4	*F*	0.335	0.859	0.906
	*p*	0.891	0.512	0.481

* Statistically significant (*p* < 0.05).

### 3.5 Isodose Line Distribution

**FIGURE 7:**
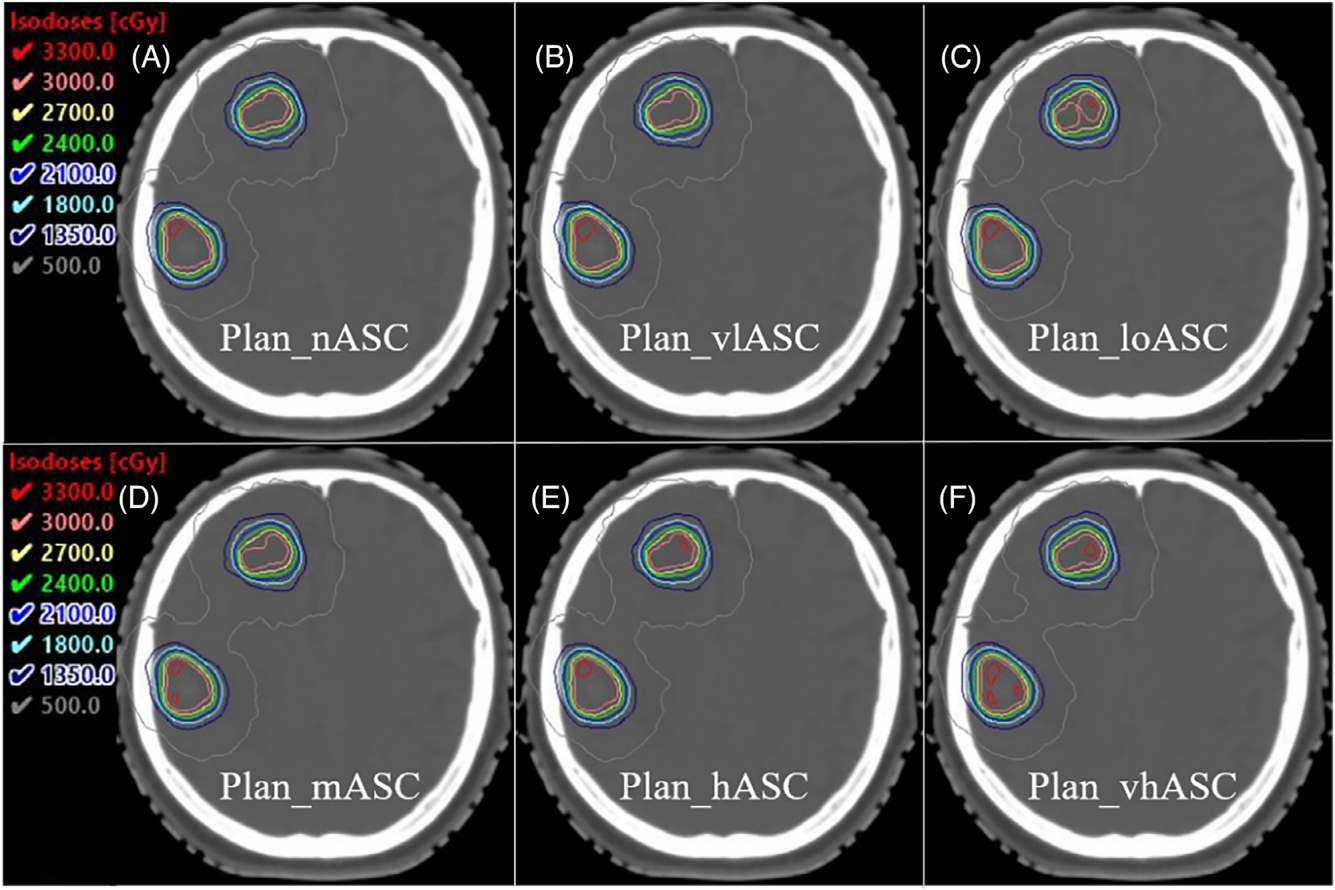
Example of isodose distributions for a representative case under different ASC strategies (axial view), representative axial isodose distributions for a single case under different ASC strategies. The prescription isodose (27 Gy, 100%) is shown as the yellow line, and the 50% isodose (13.5 Gy) is the blue line. Note the high degree of similarity in target coverage and OAR sparing, with only minor variations in the high‐dose region shape.

Visual assessment confirmed that all plans met the clinical PTV coverage and OAR sparing requirements, with highly similar overall dose distributions. Minor discernible differences were confined to precise contours of higher‐dose regions (e.g., 3000 cGy isodose) and maximum dose point locations within the PTV (Figure 7, representative case) without clinically unacceptable hotspots or cold spots.

## 4 DISCUSSION

This study systematically investigated the comprehensive impact of different ASC stratification strategies on the dosimetric quality, plan complexity, and treatment delivery efficiency of HyperArc™ SRS plans for intracranial oligometastases. The primary finding was that varying ASC strengths did not significantly compromise key dosimetric metrics, including the PTV CI and GI indices, OAR sparing, and plan deliverability (as assessed by gamma passing rates). Although most plan complexity indices (SN, MCS, and ALT) also demonstrated no statistically significant intergroup variations, a discernible trend toward improved AI was observed with moderate‐to‐high ASC strengths. Critically, ASC application, particularly at these strengths, significantly reduced the total MUs, thereby indicating a clear clinical value in enhancing treatment efficiency without a trade‐off in dosimetric plan quality.

Previous studies on conventional VMAT have demonstrated that higher ASC settings generally reduce MLC modulation complexity.^15^, ^26^ Saroj et al.,^15^ for instance, noted that “moderate” ASC strength could reduce complexity with minimal plan quality compromise, whereas “very high” strength might be preferable for maximal simplification. MU reduction is a recognized potential benefit of ASC in VMAT,^7^, ^16^ although MUs are multifactorial in nature. Aperture smoothing by ASC typically improves deliverability and reduces the sensitivity to MLC positional errors, which are crucial in complex plans.^16^, ^27^

The impact of ASC on the dosimetric quality in conventional VMAT is site‐specific and strength‐dependent.^7^, ^27^ Takaaki et al.^27^ found that ASC (including “very high” strength) reduced MLC complexity without negative DVH impact in prostate cancer. Zhu et al.^28^ recommended “low” or “moderate” ASC for cervical cancer to improve complexity and reduce MUs. Our study on intracranial oligometastases with HyperArc found a non‐significant impact of ASC strength on PTV and OAR macro‐dosimetry, partly aligning with the findings of Takaaki et al.^27^ ASC can be optimized without significant dosimetric compromise. Although trends suggested slight GI and D_2cm_ improvements with “moderate” ASC and a minor adverse CI effect with “very high” ASC; these were clinically acceptable and non‐significant. Crucially, ASC did not systematically impair OAR protection.

Our findings for the automated HyperArc SRS framework differ from those of conventional VMAT studies. ASC did not significantly affect the SN, MCS, or ALT in our study, unlike reports for conventional VMAT, where ASC effects can be pronounced; this may be attributed to HyperArc's integrated optimization engine. This engine, tailored for SRS demands (e.g., steep gradients and small target precision), possibly incorporates inherent complexity controls, such as preconfigured noncoplanar arc geometries and internal constraints on modulation designed to achieve steep dose gradients, which may attenuate the more dramatic effects of ASC observed in conventional VMAT. Nevertheless, we observed that certain ASC settings (e.g., moderate/high) demonstrated improved AI compared to ASC‐off, suggesting that ASC can still refine the MLC aperture regularity within HyperArc. This implies that the smoothing effect of ASC persists, although its influence on traditional complexity metrics might be attenuated by HyperArc's intrinsic optimization.

Although MU reduction is a recognized benefit of the ASC in conventional VMAT, its application in the highly optimized HyperArc SRS framework still yields significant efficiency gains. Specifically, high‐strength ASC (Plan_hASC) yielded significantly lower mean MUs than ASC‐off (*p* = 0.005) and was also lower (though not significantly) than very‐high‐strength ASC. This indicates that applying high‐strength ASC within the standard HyperArc workflow can effectively reduce MUs, potentially shortening treatment times without substantially affecting dosimetric quality or deliverability. This offers a simple and effective optimization pathway for clinical practice that is particularly relevant for high‐throughput centers.^5^, ^13^ Furthermore, subtle isodose distribution changes suggest that ASC might influence microdosimetric characteristics via small‐field effects and aperture smoothing, warranting further investigation.^7^, ^14^

The novelty of this study lies in its systematic evaluation of standard ASC stratification within the automated HyperArc workflow for intracranial oligometastases, highlighting its efficiency. Previous limited work combining ASC and HyperArc often used modified strategies with extra manual steps (e.g., “DCA‐dose‐first” with high‐priority ASC).^29^ Although these have demonstrated positive results (e.g., improved small‐field dosimetry and reduced MUs), they are operationally complex. Our findings suggest that the direct application of standard ASC settings (especially high strength) in the HyperArc optimizer can achieve significant efficiency gains, aligning better with HyperArc's design for automation and simplified workflows.^14^, ^16^ This is particularly valuable because it suggests that efficiency gains can be achieved without adding manual complexity to the planning process.

This study has several limitations. Its single‐center retrospective design with a small sample (*n* = 17) of patients with 1–3 small intracranial oligometastases (<25 cm) may limit the generalizability of our findings, particularly for more complex cases (e.g., >3 or larger lesions). Future multicenter collaborations would be beneficial to validate these findings across different clinical environments and patient cohorts. Furthermore, the results were based on a standard 120‐leaf MLC, and different MLC types yielded varied outcomes. Future research should, therefore, focus on larger, diverse, multicenter cohorts using different hardware and TPS versions. Ultimately, prospective trials correlating ASC‐optimized HyperArc plan characteristics with clinical outcomes such as local control and neurotoxicity are essential to completely evaluate the clinical value of this synergy.

Despite these limitations, this study provides important initial evidence for the application of ASC strategies in HyperArc therapy for intracranial oligometastases, affirming its role in optimizing delivery efficiency while maintaining dosimetric quality.

## 5 CONCLUSION

The application of different ASC strengths in HyperArc™ stereotactic radiosurgery for intracranial oligometastases does not cause clinically significant adverse effects on plan dosimetric quality, including target coverage, OAR sparing, and deliverability. However, ASC stratification significantly optimizes treatment delivery efficiency, primarily by reducing the total number MUs. High ASC strength was more effective in lowering MUs than ASC‐off and very high ASC strength. Considering the trend toward improved AI and significant efficiency gains, prioritizing moderate or high ASC strengths is recommended for HyperArc plan optimization in such cases. This strategy can enhance radiotherapy workflow efficiency while ensuring high quality and precise dose distribution, potentially improving patient experience and resource utilization. Future studies should validate these findings in larger and more diverse populations and explore their impact on clinical outcomes.

## AUTHOR CONTRIBUTIONS

**Huipeng Meng**: Supervision; conceptualization; experimental design, and writing of the original draft. **Yanlong Zhang**: Data curation; formal analysis, and conceptualization. **Xinrui Wang**: Resources; technical support. **Pengfei Liu**: Data curation and validation. **Weihua Zhu**: Technical support software. **Shixiong Huang**: software. **Yining Yang**: supervision; writing—review, and editing.

## CONSENT FOR PUBLICATION

The need for consent for publication was waived by the Institutional Review Board because of the retrospective nature of the study and the use of anonymized data.

## CONFLICT OF INTEREST DECLARATION

The authors declare that they have no competing interests.

## ETHICS APPROVAL AND CONSENT TO PARTICIPATE

This retrospective study was conducted according to the ethical guidelines of the Declaration of Helsinki. The study protocol was approved by the Science and Technology Ethics Committee of Tianjin First Central Hospital (TFCH) (Approval No. 20241031‐1), which waived the requirement for patient informed consent because of the retrospective nature of the analysis and the utilization of fully anonymized data.

## Data Availability

The data that support the findings of this study are available from the corresponding author upon reasonable request.
